# Freestanding Polymer Metasurface Supporting Higher‐Order Optical Resonances for Strong Field Enhancement in TMD Monolayers

**DOI:** 10.1002/smll.202513320

**Published:** 2026-05-17

**Authors:** Chih‐Zong Deng, Sunhao Shi, Chun‐Hao Chiang, Mu‐Hsin Chen, Shuaicheng Liu, Haruyuki Sakurai, Jui‐Han Fu, Kuniaki Konishi, Masanobu Iwanaga, Vincent Tung, Ya‐Lun Ho

**Affiliations:** ^1^ Research Center for Electronic and Optical Materials National Institute for Materials Science (NIMS) Tsukuba Ibaraki Japan; ^2^ Department of Chemical System Engineering Graduate School of Engineering The University of Tokyo Bunkyo Tokyo Japan; ^3^ Department of Physics Graduate School of Science The University of Tokyo Bunkyo Tokyo Japan; ^4^ Institute For Photon Science and Technology Graduate School of Science The University of Tokyo Bunkyo Tokyo Japan

**Keywords:** light–matter interaction, membrane, suspended metasurface, transition metal dichalcogenide monolayer

## Abstract

Enhancing light–matter coupling in two‐dimensional (2D) semiconductors, such as transition metal dichalcogenide monolayers, remains a central challenge in nanophotonics due to their atomic thickness, which limits their interaction volume with light. Here, we demonstrate that higher‐order optical resonances, including photonic guided modes (GMs) and quasi‐bound states in the continuum (quasi‐BICs) supported by a freestanding metasurface, provide exceptionally strong surface field enhancement, enabling efficient coupling with a tungsten disulfide (WS_2_) monolayer. Triangular‐lattice polymer patterns on silicon nitride membranes are fabricated to realize these higher‐order modes. Simulations reveal that second‐order modes possess optimal surface electric‐field distributions that strongly overlap with the overlying WS_2_ monolayer, significantly outperforming their first‐order counterparts. Photoluminescence (PL) measurements confirm a remarkable PL enhancement factor of 193 for the second‐order GM, over an order of magnitude greater than that of the first‐order modes. These results establish higher‐order modes in freestanding metasurfaces as a promising route to engineer light–matter interactions in 2D semiconductors for advanced nanophotonic and quantum photonic applications.

## Graphical abstract

A freestanding polymer metasurface is developed to enhance light emission from atomically thin semiconductors. By supporting higher‐order optical resonances that concentrate electromagnetic fields at the surface, the platform significantly improves coupling between light and WS_2_ monolayer. This approach enables stronger photoluminescence without complex material processing, offering a scalable route for integrating two‐dimensional materials into advanced photonic devices.

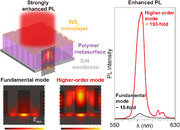

## 1 Introduction

Two‐dimensional (2D) transition metal dichalcogenides (TMDs), such as WS_2_ and MoS_2_ monolayers, have emerged as a central platform for advancing next‐generation optoelectronic and quantum technologies [1, 2]. Their atomically thin nature provides unique opportunities for extreme miniaturization and integration, while their strong excitonic resonances and valley‐selective optical selection rules open pathways toward exciton‐based devices. These features have motivated intense research into TMD‐based photodetectors, light‐emitting devices, and quantum emitters, positioning them as key building blocks for future information and communication technologies [3, 4, 5, 6, 7, 8]. However, a major bottleneck arises from the intrinsically weak interaction volume between light and monolayer semiconductors. Because TMD monolayers are atomically thin, their absorption and emission cross‐sections are small compared to bulk semiconductors, severely limiting their efficiency when integrated into optical devices. Overcoming this limitation requires carefully engineered photonic environments that can amplify and control light–matter interactions at the nanoscale.

Nanophotonic resonators and metasurfaces offer an attractive solution to this challenge. By confining electromagnetic fields into subwavelength regions and enhancing their intensity, resonant photonic structures can compensate for the low optical volume of TMD monolayers. This strategy has already been demonstrated in various contexts, including photonic crystals [4], plasmonic nanoantennas [6], and dielectric metasurfaces [9]. Among these approaches, dielectric metasurfaces are particularly promising due to their compatibility with large‐area active areas, low optical losses, and ability to sustain a rich set of resonant modes with tailored spectral and spatial characteristics.

In this context, bound states in the continuum (BICs) supported by dielectric metasurfaces have recently emerged as an especially powerful concept for enhancing light–matter interactions [10, 11]. BICs are non‐radiating states that remain perfectly confined within photonic systems, despite being embedded in the radiation continuum. When perturbed by symmetry breaking or fabrication imperfections, they transform into quasi‐BICs with extremely high but finite quality (Q) factors. These quasi‐BIC metasurfaces produce strong field confinement and narrow resonances, enabling applications in lasing [12, 13, 14], sensing [15], nonlinear optics [16], and quantum light generation [17]. The ability of quasi‐BIC metasurfaces to concentrate light into subwavelength regions with minimal radiative loss makes them suited for coupling with atomically thin materials such as TMD monolayers [18, 19, 20, 21, 22, 23, 24, 25, 26, 27].

A critical design consideration in enhancing light–matter interaction is whether the metasurface is supported on a substrate or suspended as a freestanding structure. Substrate‐supported structures are easier to fabricate and integrate but suffer from several limitations: the lower refractive index contrast between the substrate and the nanostructure often leads to radiation leakage into the substrate, reducing the achievable Q‐factors; furthermore, the substrate can perturb the field distribution, reducing the overlap of resonant modes with surface‐bound emitters [28, 29]. Recent works have demonstrated that membrane‐based metasurfaces can effectively suppress substrate‐induced leakage [30, 31] and enable access to higher‐order resonances [32] with improved confinement and symmetry. In particular, these studies highlight the role of vertical symmetry restoration and high index contrast in stabilizing guided‐mode (GM) resonances and quasi‐BIC states. In contrast, freestanding membranes eliminate substrate leakage pathways and allow for symmetric light confinement on both sides of the structure [18, 19, 20, 33].

Silicon nitride (SiN) membranes with comparable thickness and lateral dimensions are known to exhibit high intrinsic tensile stress, which mechanically stabilizes the suspended structure and suppresses buckling or wrinkling after release. The tensile stress promotes global planarity and minimizes membrane curvature, even for relatively large, suspended areas. As a result, such membranes typically maintain excellent structural flatness and uniform stress distribution, which is essential for preserving optical symmetry and high‐Q resonances [34, 35]. Furthermore, substrate removal restores vertical symmetry and suppresses additional radiation channels, enabling higher Q‐factors in photonic crystal slabs [18, 19, 20]. The freestanding configuration also maximizes refractive index contrast and enhances near‐surface field confinement, which is particularly advantageous for coupling to monolayer 2D materials. Within this mechanically and optically robust freestanding platform, the choice of patterned material becomes a key factor in determining device functionality and fabrication practicality. The utilization of polymer as photonic device material provides significant advantages in terms of fabrication efficiency, structural quality [31, 36, 37, 38], and sustainability. Unlike traditional dielectric metasurfaces that require complex multi‐step procedures including material deposition, hard‐masking, and reactive ion etching, the polymer‐based approach collapses the workflow into three primary steps: spin‐coating, exposure, and development. By repurposing the resist as the final device material, etching‐related defects such as material redeposition and geometric perturbations are eliminated, ensuring high‐fidelity nanopatterns whose precision is limited only by the initial lithography step. Although polymers possess a lower refractive index compared to traditional semiconductors like silicon, the implementation of a freestanding architecture maximizes the index contrast with the surrounding air.

Most prior studies have focused on exploiting first‐order modes. These fundamental modes, which typically concentrate fields inside the photonic slab, have been used to enhance spontaneous emission, exciton–polariton formation, and nonlinear optics in TMDs [18, 21, 22, 23, 24, 25, 26, 27]. However, reports on higher‐order modes remain limited [13, 14, 39]. Because of their distinct field distributions, second‐order modes exhibit stronger field localization near the surface region, thereby improving coupling efficiency with material placed on the surface. Such surface‐localized resonances, which exhibit strong field enhancement on the top surface, are especially advantageous for TMD monolayers, enabling efficient coupling where the material is placed. Despite this compelling potential, systematic investigations into the role of higher‐order modes for enhancing TMD emission remain scarce.

Here, we experimentally demonstrate that second‐order modes supported by a freestanding metasurface—comprising a polymer hole‐array slab on a SiN membrane—enable dramatically stronger photoluminescence (PL) enhancement in WS_2_ monolayers on top of the metasurface. Through field enhancement simulations and PL measurements, we identify the field distributions of second‐order modes and confirm their decisive role in enhancing the field on the top surface. Our results demonstrate second‐order modes in freestanding metasurfaces as a powerful new design principle for boosting light–matter interaction in 2D materials. This approach can offer a general platform for engineering strong excitonic effects, nonlinear responses, and quantum optical functionalities, opening new directions for hybrid photonic–2D material systems.

## 2 Results and Discussion

**FIGURE 1:**
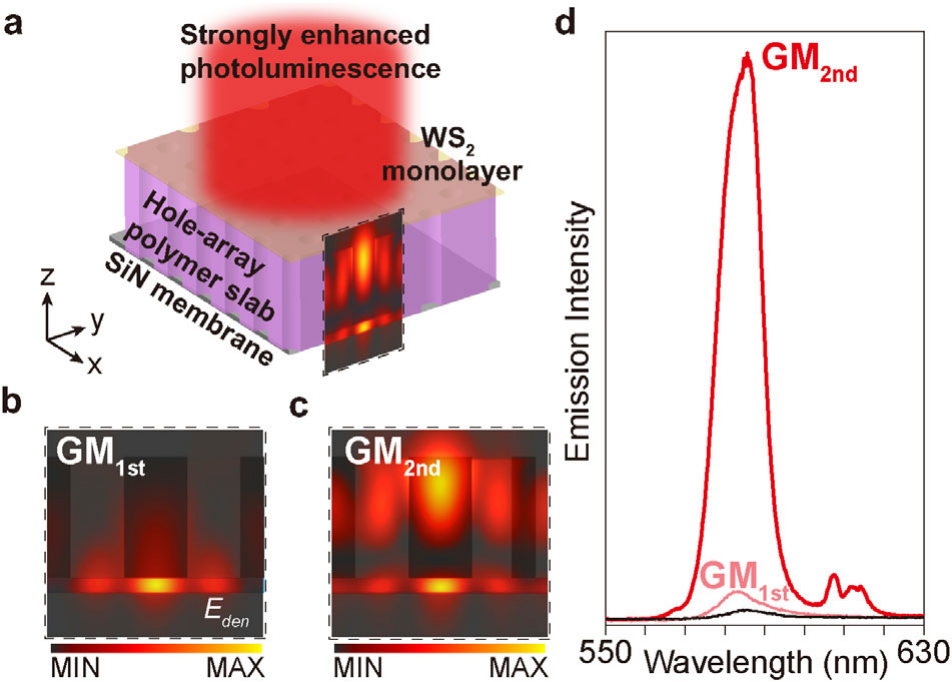
Photoluminescence (PL) from a tungsten disulfide (WS_2_) monolayer strongly enhanced by a freestanding metasurface supporting second‐order guided mode (GM), which provide intense surface field confinement and efficient exciton–photon coupling. (a) Schematic illustration of the concept: A WS_2_ monolayer is placed on a freestanding metasurface, which is a hole‐array polymer slab on a silicon nitride (SiN) membrane. Simulated electric energy density *E_den_* distribution at (b) first‐order GM and (c) second‐order GM in *yz*‐plane. (d) PL spectra of the WS_2_ monolayer under first‐order GM (light red curve), second‐order GM (red curve), and on an unstructured membrane (black curve).

Figure 1 illustrates the freestanding metasurface designed to strongly enhance the field near the top surface, thereby significantly enhancing the PL of a TMD monolayer at the surface. This enhancement is achieved by leveraging the second‐order modes. As shown in Figure 1a, the proposed device consists of a freestanding metasurface—a polymer hole‐array slab on a SiN freestanding membrane. This specific geometry is critical for supporting the desired second‐order modes. The simulated electric energy density *E_den_* profiles of the proposed freestanding metasurface, as shown in Figure 1b,c, visually confirm the strong field confinement. The second‐order mode shows a strong field enhancement near the top surface of the metasurface, where the monolayer is located. This strong overlap between the field and the exciton‐rich region of the monolayer is essential for achieving efficient exciton‐photon coupling. In contrast, the first‐order mode exhibits a weaker and more delocalized field at the top surface, leading to a less effective interaction. Figure 1d shows the PL spectrum of the WS_2_ monolayer on the second‐order modes metasurface (red curve), which shows a remarkable PL enhancement—a dramatic increase in intensity compared to the PL from the same WS_2_ monolayer on the first‐order modes metasurface (light red curve) or the WS_2_ monolayer on the unstructured membrane (black curve). The strongly enhanced PL confirms that the second‐order modes significantly boost the radiative efficiency of the WS_2_ monolayer, a direct consequence of the strongly enhanced field and efficient exciton‐photon coupling.

**FIGURE 2:**
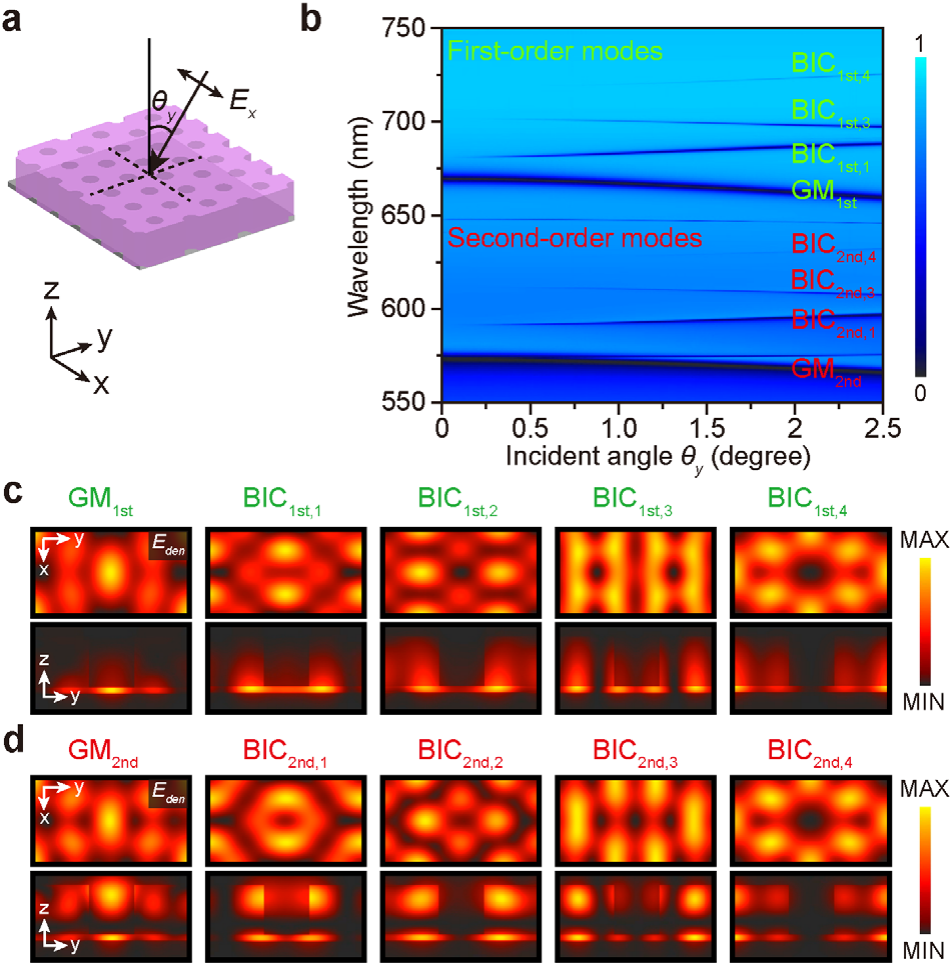
Freestanding metasurface engineered to support second‐order GMs and quasi‐BICs with strong field enhancement at the top surface. (a) Schematic diagram of the membrane metasurface, consisting of a triangular‐lattice air‐hole array patterned in polymer resist with a lattice period *P* = 560 nm, a hole diameter *D* = 280 nm, and a thickness *T* = 400 nm on a 50 ‐nm‐thick SiN membrane. (b) Simulated angle‐resolved transmittance spectra under *x*‐polarized illumination along the *y*‐direction, showing GMs and quasi‐BICs. Electric energy density *E_den_* distributions for the (c) first‐order and (d) second‐order modes, the incident angle *θ* = 2.5°.

Figure 2a shows a schematic diagram of the proposed freestanding metasurface, consisting of a triangular‐lattice air‐hole array patterned in polymer resist with a lattice period *P* = 560 nm, a hole diameter *D* = 280 nm, and a thickness *T* = 400 nm on a 50‐nm‐thick SiN freestanding membrane. This freestanding metasurface is designed to support GMs and BICs, with second‐order modes in particular providing a strong field enhancement for the light–matter interactions with a WS_2_ monolayer. The simulated angle‐resolved transmittance spectra of the freestanding metasurface are shown in Figure 2b. The light is *x*‐polarized illumination along the *y*‐direction, calculated using rigorous coupled‐wave analysis (RCWA) to characterize the structure. The incident angle *θ_y_* is varied between 0° and 2.5° to resolve symmetry‐protected BICs expected at the Γ point. At normal incidence, the spectrum exhibits vanishing linewidths for BIC modes, allowing a clear distinction from GMs. In the longer wavelength range, the four first‐order modes are identified, including one GM (GM_1st_) and three BICs (BIC_1st,1_, BIC_1st,3_, BIC_1st,4_). In the shorter wavelength range, four second‐order modes are identified, including one GM (GM_2nd_) and three BICs (BIC_2nd,1_, BIC_2nd,3_, BIC_2nd,4_). In addition, BIC_1st,2_ and BIC_2nd,2_ are accessible under *x*‐tilted illumination (*θ_x_*), as shown in Figure S1. In practice, perfect BICs transform into quasi‐BICs due to inevitable radiation leakage arising from fabrication imperfections, finite‐size effects, and nonzero incident angles. Nevertheless, they maintain exceptionally high Q‐factors, enabling strong light confinement. The spatial distributions of the *E_den_* for the first‐order and second‐order modes at *θ_y_* = 2.5° are shown in Figure 2c,d, respectively. The top panels show the in‐plane (*xy*‐plane) field profiles at the middle of the SiN membrane, while the bottom panels show the out‐of‐plane (*yz*‐plane) cross‐sections through the unit cell. The *xy*‐plane field profiles confirm that the first‐ and second‐order modes share similar in‐plane symmetries, consistent with their transverse electric nature (Figure S2). In contrast, the *yz*‐plane field distributions highlight a distinct difference in vertical mode order: the first‐order mode exhibits a single antinode confined within the SiN membrane, whereas the second‐order mode features two antinodes—one within the membrane and another near the polymer–air interface. This strong field enhancement on the top surface highlights the potential of second‐order modes as an ideal environment for coupling with TMD monolayers, enabling efficient PL enhancement and exciton–photon interactions.

**FIGURE 3:**
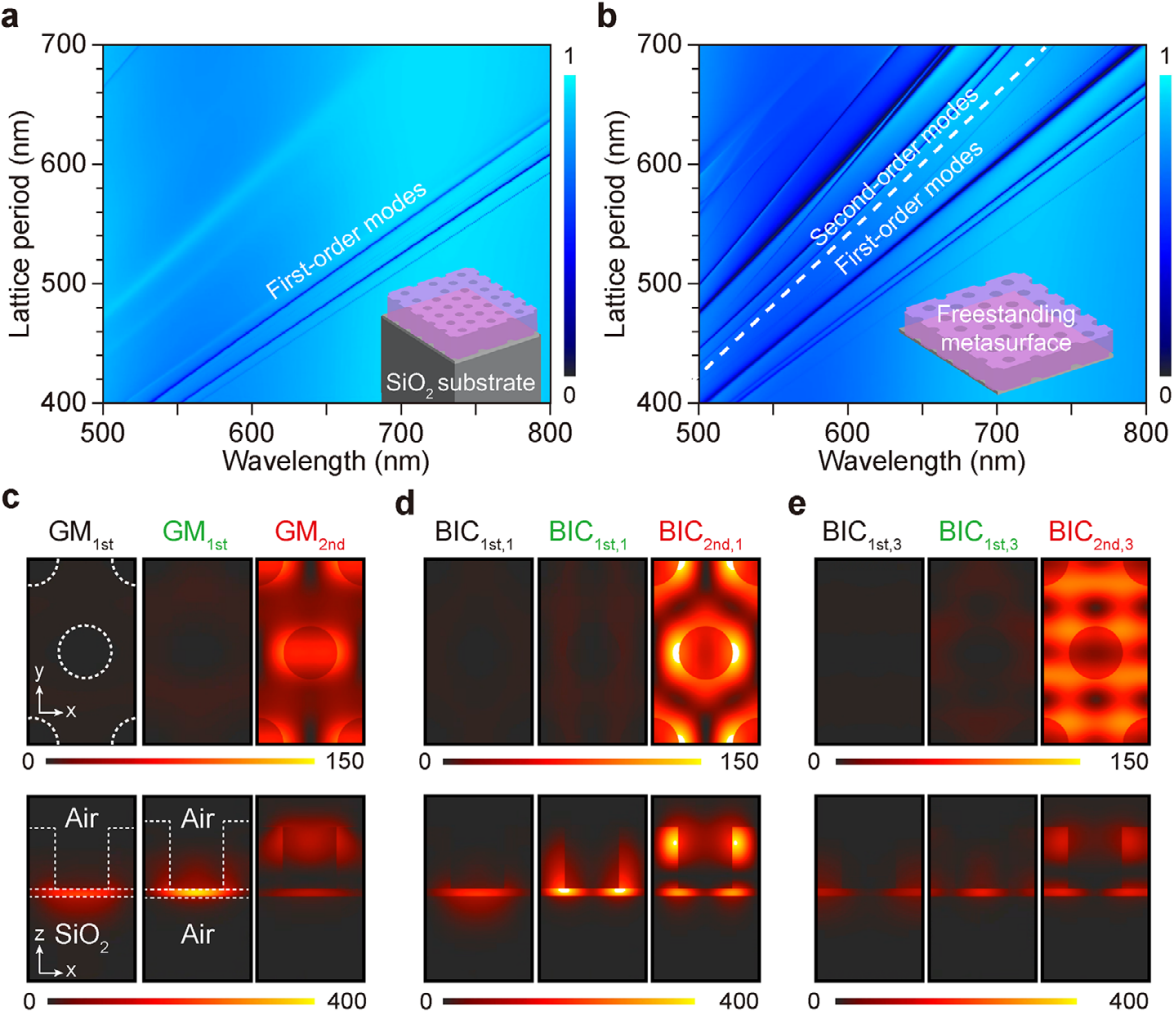
Strong field enhancement on the top surface of the freestanding metasurface via second‐order modes. Simulated transmittance spectra as a function of lattice period for (a) substrate‐based metasurface and (b) freestanding metasurface. Electric energy density *E_den_* distributions in the *xy*‐plane at the top surface and *xz*‐plane for (c) GM, (d) BIC_1,_ and (e) BIC_3_ in both the substrate‐based metasurface and freestanding metasurface. The incident light is *x*‐polarized with an incident angle of 2.5° along the *y*‐direction.

To highlight the importance of the freestanding architecture for accessing second‐order modes with strong surface field confinement, Figure 3 compares the substrate‐supported metasurface and the freestanding metasurface, further contrasting the field enhancement of first‐ and second‐order modes. Figure 3a,b shows the simulated transmittance spectra, both at an incident angle *θ_y_* = 2.5°, under varying lattice period for polymer hole‐array slabs on a silicon dioxide (SiO_2_) substrate and the same slab on a SiN freestanding membrane, respectively. In both cases, the resonance wavelengths redshift with increasing lattice period, consistent with the enlarged effective optical path. Notably, the resonant wavelength can be readily tuned across the visible range by adjusting the lattice period, allowing precise spectral alignment with the excitonic transition of TMD monolayers to maximize light–matter interaction through exciton–photon coupling. As shown in Figure 3b, both first‐ and second‐order modes are clearly observed in the freestanding metasurface. In contrast, in the substrate‐supported metasurface (Figure 3a), the reduced vertical refractive index contrast between the polymer layer and the supporting substrate introduces additional radiation channels, which suppress the formation of second‐order GMs and quasi‐BICs. Moreover, significant field leakage into the substrate leads to resonance attenuation, resulting in reduced transmittance contrast.

Figure 3c–e presents the spatial distribution of the *E_den_* for the GM, BIC_1,_ and BIC_3_ at *θ_y_* = 2.5°. The resonance wavelengths of the modes were tuned near the excitonic emission peak wavelength of WS_2_ (∼620 nm) by setting the lattice periods *P* to 480 and 560 nm for the first‐ and second‐order modes, respectively. The top panels display the in‐plane (*xy*‐plane) field profiles at the top surface, while the bottom panels show the out‐of‐plane (*xz*‐plane) cross‐sections through the unit cell. For the substrate‐supported case (left panel of Figure 3c–e), although the field is strongly confined within the SiN membrane, the enhancement at the top surface is reduced by approximately two orders of magnitude, resulting in a weak field localized at the top surface. All modes in the substrate‐supported case exhibit significantly weaker field enhancement compared to those in the freestanding metasurfaces (middle and right panel), revealing pronounced leakage into the substrate.

In contrast, the freestanding metasurfaces enable stronger field enhancement near the top surface. The first‐order modes show slightly stronger enhanced surface fields compared to the substrate‐supported metasurface, while the second‐order modes exhibit even stronger enhancement. Comparing the surface field enhancement among first‐ and second‐order modes, the maximum *E_den_* for GM_2nd_, BIC_2nd,1_, and BIC_2nd,3_, are 113, 166, and 95, respectively—much larger than their first‐order counterparts GM_1st_ (4), BIC_1st,1_ (5), and BIC_1st,3_ (7). This enhancement arises because the antinodes of the field in second‐order modes coincides with the near top surface (bottom panel). In summary, substrate‐induced leakage suppresses the formation of second‐order modes, whereas the freestanding configuration preserves them. Owing to their field distributions, the second‐order modes provide substantially stronger surface field enhancements than the first‐order modes, directly benefiting light–matter interactions with TMD monolayers.

**FIGURE 4:**
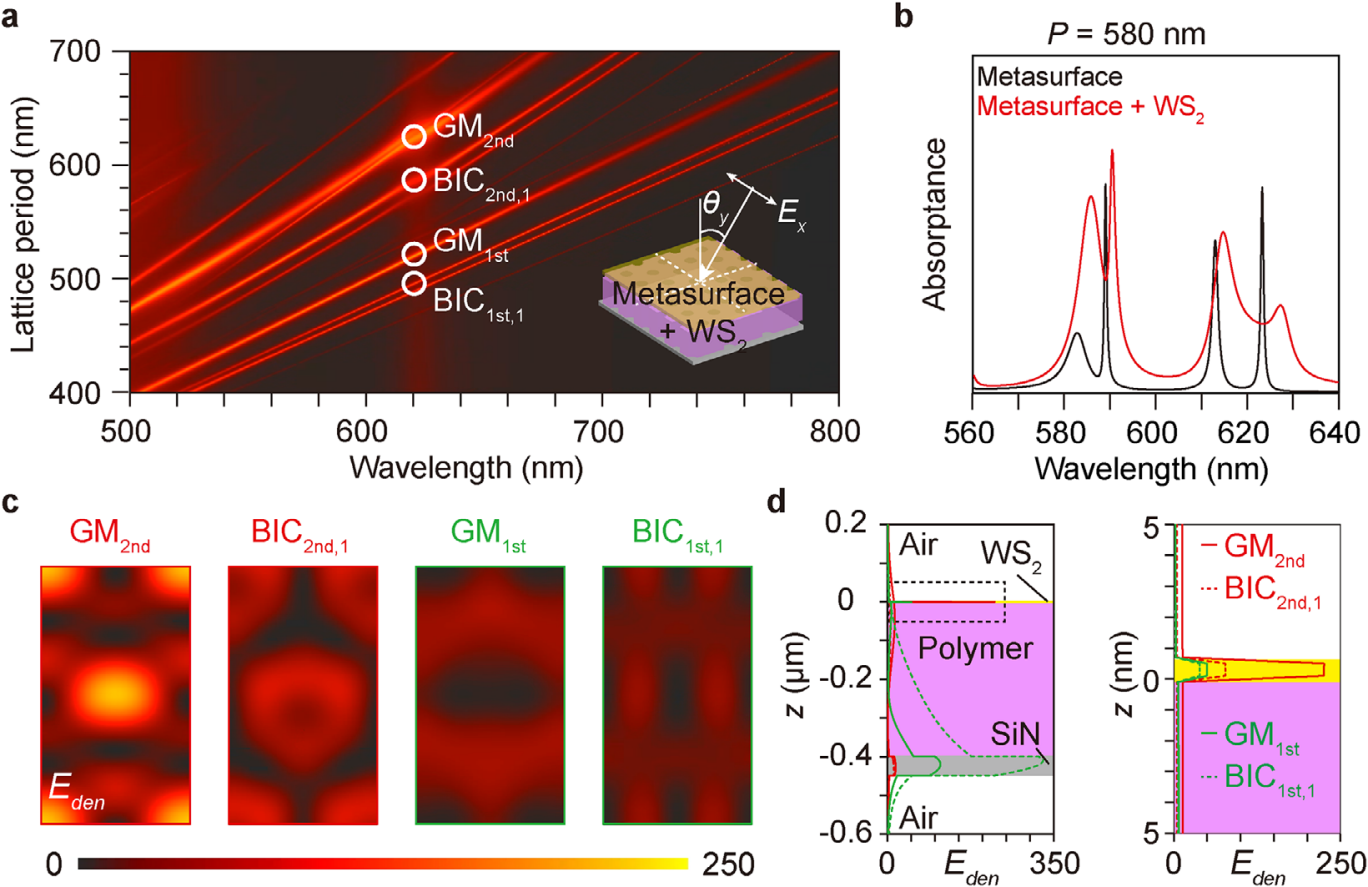
Coupling of an WS_2_ monolayer to a freestanding metasurface. (a) Simulated absorption spectra as a function of lattice period for a freestanding metasurface. (b) Simulated absorption spectra of freestanding metasurface before and after WS_2_ monolayer transferred. (c) *E_den_* distributions in the *xy*‐plane at the middle of WS_2_ monolayer for GM_2nd_, BIC_2nd,1_, GM_1st_, and BIC_1st,1_ in a freestanding metasurface. (d) *E_den_* distributions along the *z*‐direction across the entire device and a magnified view of specially within the WS_2_ layer, highlighting the localized field enhancement. The incident light is *x*‐polarized with an incident angle of 2.5° along the *y*‐direction.

Figure 4 illustrates the coupling between the WS_2_ monolayer and the proposed freestanding metasurface. Figure 4a shows the simulated absorption spectra for the integrated device under varying lattice periods, confirming that all optical modes persist following the transfer of the WS_2_ monolayer (Figure 4a). These resonances exhibit a characteristic redshift due to the high refractive index of the WS_2_ (Figure 4b). It is noteworthy that the Q‐factor undergoes degradation upon integration with WS_2_, particularly for the quasi‐BICs, which are highly sensitive to local environmental changes and damping. For a metasurface with *P* = 580 nm, the Q‐factor of the BIC_1st,1_ decreases from 398 (bare) to 100 (with WS_2_). In contrast, the first‐order guided mode (GM_1st_) proves to be significantly more robust, with its Q‐factor shifting only from 111 (bare) to 92 (with WS_2_). Note that the simulated Q‐factors were calculated at a fixed incident angle of 2.5°. This demonstrates that while quasi‐BICs offer higher theoretical Q‐factors, they are heavily dampened by the large absorption of the WS_2_ layer.

To compare the field enhancement of different optical modes at the peak absorption wavelength of WS_2_ (620 nm), the metasurface periods were tuned to align each resonance with this wavelength, as indicated in Figure 4a. The resulting *E_den_* distributions within the WS_2_ layer are presented in Figure 4c. These results indicate that second‐order modes provide greater field enhancement within the WS_2_ layer than first‐order modes. Specifically, the GM_2nd_ exhibits a maximum enhancement factor of 227, which is more than four times the enhancement of 50 observed for the GM_1st_. This confirms that while the WS_2_ layer perturbs the optical resonances, light–matter interaction is nonetheless significantly amplified, particularly for the GMs, which demonstrate lower sensitivity to environmental perturbations and material loss. One‐dimensional *E_den_* profiles (Figure 4d) further confirm that the field is strongly confined within the WS_2_ layer for second‐order modes (GM_2nd_ and BIC_2nd,1_). In contrast, the first‐order mode (GM_1st_ and BIC_1st,1_) primarily localizes the field within the SiN layer rather than the WS_2_ monolayer, resulting in poor coupling and lower enhancement factors.

**FIGURE 5:**
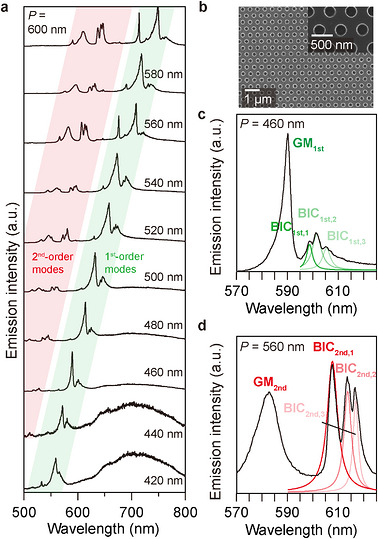
Demonstration of both first‐order and second‐order GMs and quasi‐BICs by freestanding metasurfaces. (a) PL spectra of the freestanding metasurfaces as a function of lattice period. (b) Scanning electron microscopy (SEM) images of the freestanding metasurface with a lattice period *P* = 520 nm. (c,d) PL spectra highlighting the spectral regions corresponding to the first‐order (*P* = 460 nm) and second‐order (*P* = 560 nm) resonances, respectively.

The designed freestanding metasurface was fabricated for experimental characterization of its optical properties. Figure S1 displays the angle‐resolved transmission for the fabricated freestanding metasurface (*P* = 560 nm). As predicted, the BICs exhibit a narrowing linewidth that approaches zero at normal incidence. In contrast, the GMs maintain strong resonances at normal incidence. Since the SiN membrane used in this work exhibits defect‐related states, a broad‐band PL emission is observed under optical pumping (Figure S4), allowing the investigated GMs and quasi‐BICs to be coupled to this PL band. The 488 nm CW laser used for PL excitation is linearly polarized. Prior to the measurements, the polarization axis of the laser beam was confirmed by using a polarizer. To ensure consistency with the simulation, the metasurface was mounted on a rotation stage under an optical microscope (OM). We aligned the *x*‐axis of the metasurface parallel to the pre‐determined linear polarization direction of the 488 nm pump beam. Figure 5a presents experimental PL spectra from freestanding metasurfaces with varying lattice periods *P* (420 to 600 nm) before the WS_2_ monolayer was transferred. PL spectra reveal multiple emission peaks corresponding to optical resonances. As expected from simulations, the resonance wavelengths redshift with increasing lattice period due to the enlarged optical path. For the lattice period of 420 nm, a group of resonances appears near a wavelength of 550 nm, corresponding to first‐order modes, and shifts to a wavelength of 750 nm for a 600‐nm lattice period. At 460, 480, and 500‐nm periods, the first‐order modes occur around 620 nm, overlapping with the exciton emission wavelength of WS_2_. In addition, second‐order modes emerge at shorter wavelengths (at 510 nm for a 440‐nm lattice period). For 560, 580, and 600‐nm periods, the second‐order modes overlap with the exciton emission wavelength of WS_2_. For GMs, the GM_1st_ at 590.2 nm with a Q‐factor of 138 (460 nm lattice period) and GM_2nd_ at 582.6 nm with a Q‐factor of 55 (560 nm lattice period) near the exciton emission wavelength WS_2_. The PL spectra with a narrow range to highlight the first‐ and second‐order quasi‐BICs are shown in Figure 5c,d. For a 460‐nm period (Figure 5c), the multi‐peak group is decomposed into Lorentzian components corresponding to BIC_1st,1_, BIC_1st,2_, and BIC_1st,3_ at 598.7, 601.5, and 605.4 nm with Q‐factors of 214, 143, and 162, respectively. For a 560‐nm lattice period (Figure 5d), the first‐order group decomposes into BIC_2nd,1_, BIC_2nd,2_, and BIC_2nd,3_ at 607.7, 613.7, and 616.9 nm with Q‐factors of 122, 173, and 251, respectively. These results experimentally demonstrate that the freestanding metasurface architecture can support both first‐ and second‐order optical modes across the visible spectrum by tuning the lattice period, providing a versatile platform for enhancing light–matter interactions with 2D materials such as TMD monolayers.

**FIGURE 6:**
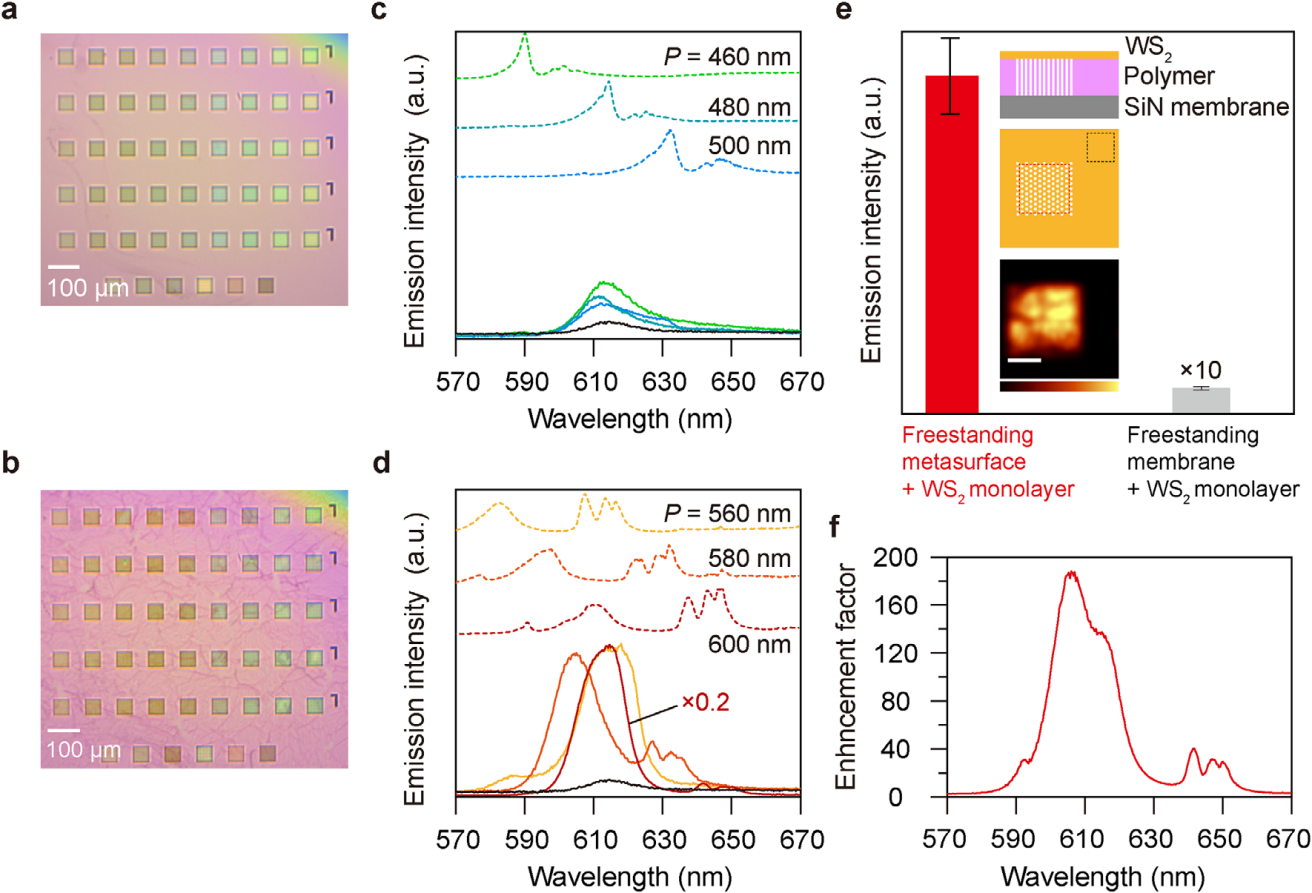
Enhanced PL emission from WS_2_ monolayers via first‐order and second‐order resonances in freestanding metasurfaces. (a, b) Optical microscope images of the freestanding metasurfaces with lattice periods of *P* = 420, 440, 460, 480, 500, 520, 540, 560, and 580 nm in the top five rows, and *P* = 300, 400, 500, 600, 700, and 800 nm in the bottom row, shown (a) before and (b) after transfer of the WS_2_ monolayer. (c, d) PL spectra of WS_2_ monolayers coupled with (c) first‐order modes (*P* = 460, 480, and 500 nm) and (d) second‐order modes (*P* = 560, 580, and 600 nm). The PL spectrum of WS_2_ on an unstructured freestanding membrane (black solid curve) and the PL spectra of the bare metasurfaces (dashed curves) are provided for reference. (e) Comparison of the peak PL emission intensities for the WS_2_ monolayer on the metasurfaces versus the unstructured membrane. Error bars represent the standard deviation calculated across 16 measurement points. Inset: PL intensity mapping at the peak emission wavelength and a schematic indicating the sampled areas. The average intensities were calculated from the regions indicated by the red (metasurface) and black (unstructured membrane) dashed squares. The scale bar is 20 µm. (f) PL enhancement factor for the metasurface with a 600 nm period, defined as the ratio of the PL intensity on the metasurface to that on the unstructured membrane, demonstrating the superior performance of the second‐order guided mode.

To verify the PL enhancement arising from the enhanced fields of the optical modes, WS_2_ monolayers were transferred onto the freestanding metasurfaces (Figure 6). OM images of the fabricated structures before and after the WS_2_ transfer are shown in Figure 6a,b, respectively. Figure 6c,d presents the PL spectra obtained from the metasurfaces supporting first‐order and second‐order modes. In Figure 6c, the metasurfaces with lattice periods *P* = 460, 480, and 500 nm support first‐order modes that spectrally overlap with the WS_2_ exciton emission band. The emission spectra from bare metasurfaces (dashed curves) and from WS_2_ on an unstructured membrane (black solid curve) are provided for reference. As shown in Figure 6c, the PL intensities from these periods show no significant differences, indicating that the first‐order modes couple only weakly to the WS_2_ monolayer. The observed minor PL enhancement primarily originates from the partially freestanding nature of the WS_2_ over the air holes, which reduces substrate‐induced quenching and nonradiative recombination, rather than from the optical resonance effects of the first‐order modes. This is further confirmed by PL mapping across large areas (Figure S5), which demonstrates consistent enhancement across the patterned regions that significantly exceeds the fluctuations in the unstructured regions. Figure 6d displays the PL intensities for metasurfaces with *P* = 560, 580, and 600 nm, which support second‐order modes overlapping with the WS_2_ emission band. For *P* = 560 nm, the PL is substantially enhanced as the second‐order quasi‐BICs spectrally align with the WS_2_ emission peak, with a secondary contribution from the second‐order GM appearing around 582 nm. At *P* = 580 nm, the PL enhancement remains high, benefiting from the simultaneous contribution of both the second‐order GM and quasi‐BIC at 605 and 630 nm, respectively. Notably, at *P* = 600 nm, the PL enhancement is significantly greater than that observed at other periods. This superior performance is attributed to the precise spectral overlap between the second‐order GM and the WS_2_ emission band, alongside the high spatial overlap between the second‐order mode's surface‐localized field and the WS_2_ monolayer. This facilitates efficient exciton–photon coupling, resulting in a markedly intensified PL peak. These experimental findings are in excellent agreement with our simulations (Figure 4), which predicted that the second‐order GM would exhibit high absorption and robustness, leading to strong PL enhancement. Figure 6e presents the average PL intensity calculated from 16 different locations on the patterned metasurface (red dashed square) compared to 16 locations on the unstructured membrane (black dashed square). The inset of Figure 6e shows the corresponding PL intensity mapping, with error bars representing the standard deviation. These results confirm the high quality of the transferred WS_2_ and validate the reported enhancement factors. The enhancement factor, defined as the ratio of PL intensity on the metasurface to that on the unstructured membrane, reaches a peak of 193 for the second‐order GM at *P* = 600 nm. Even the second‐order quasi‐BIC resonance yields a substantial enhancement factor of 84, both of which far exceed the maximum enhancement of 15 observed for the first‐order modes.

## 3 Conclusions

We have demonstrated that freestanding metasurfaces can host both first‐ and second‐ order optical modes across the visible spectrum, enabling efficient coupling with WS_2_ monolayers. While first‐order modes primarily confine fields inside the freestanding metasurface, second‐order modes provide strong field enhancement on the top surface of the freestanding metasurface that overlaps with the WS_2_ monolayer. As a result, second‐order resonances provide significantly stronger PL enhancement—exceeding one order higher than first‐order counterparts. Our experimental and simulation results demonstrate second‐order modes in freestanding metasurfaces as a powerful design principle for enhancing light–matter interactions in 2D materials such as TMD monolayer. The proposed freestanding metasurface serves as a versatile photonic platform for enhancing light–matter interaction in a broad range of 2D materials. The resonance wavelength can be readily tuned across the visible and near‐infrared spectrum by adjusting the lattice period, enabling spectral matching to the excitonic transitions of various TMDs (e.g., MoS_2_, MoSe_2_, and WSe_2_). Moreover, the strong field confinement at the top interface makes the structure compatible with other atomically thin materials, including graphene for enhanced photodetection and hBN for quantum emission applications. This scalability highlights the generality of the platform for diverse 2D material systems. Furthermore, our analysis indicates that while the dispersion behaviors of the first‐ and second‐order modes are fundamentally similar (Figure S8), the structured PL in momentum space is dictated by the radiative channels of these resonant modes. By aligning the excitonic transitions of WS_2_ with specific points in the metasurface dispersion, the optical modes provide a platform for controlling the radiation directivity [22].

Beyond PL enhancement, this approach opens new pathways for engineering strong exciton–photon coupling, nonlinear processes, and quantum optical functionalities in 2D material–based nanophotonic devices. Notably, the open‐system nature of these freestanding structures connects our findings to the emerging field of non‐Hermitian physics. Recent progress in non‐Hermitian photonics has shown that radiative loss and dissipation can lead to exceptional points (EPs), where eigenvalues and eigenstates simultaneously coalesce, enabling unconventional wave control and enhanced functionalities. EPs have been demonstrated in metasurfaces and open photonic systems [40, 41]. While our work focuses on resonances in passive structures, their open‐system nature connects to non‐Hermitian physics and suggests opportunities for future EP engineering. Such developments could further expand the utility of this platform toward active topological photonics and ultra‐sensitive sensing applications.

## 4 Methods

### 4.1 Numerical Simulations

The far‐field reflectance spectra of the freestanding metasurfaces and the near‐field electric field profiles of their resonant modes were numerically analyzed using the rigorous coupled‐wave analysis method (DiffractMOD, RSoft Design Group, USA). The simulations employed periodic boundary conditions along the *x*‐ and *y*‐directions. The complex refractive indices (n, k) for the SiN membrane, polymer resist, and WS_2_ monolayer were experimentally determined via spectroscopic ellipsometry, with the resulting dispersion data provided in Figure S6. The wavelength resolution of the simulation is 10^−5^ µm. A plane wave was used as the incident source, propagating along the z‐axis. The electric field **E** was normalized to the amplitude of the incident field. The electric energy density was calculated as *U_E_* = ½∫Re[*ε*(**r**′)]|**E**|^2^ *dV*, where **E** is the electric field, ε is the spatially dependent permittivity, and V is the volume of the simulation domain.

### 4.2 Fabrication

A triangular lattice hole‐array pattern was defined in the polymer resist on the 50 nm‐thick SiN membrane using electron‐beam lithography. The SiN membranes were provided by Norcada, Inc. The nanorod patterns were drawn on a positive electron‐beam resist (AR‐P6200 (CSAR 62), Allresist, Germany), using a high‐resolution electron‐beam‐drawing instrument (ELS‐BODEN, ELIONIX, Japan) as shown in Figure S7.

### 4.3 Preparation for WS_2_ monolayer

WS_2_ films were grown via standard chemical vapor deposition (CVD) using a one‐zone horizontal quartz tube furnace (2‐inch diameter). Tungsten trioxide (WO_3_, Sigma–Aldrich, 99.9%, 100 mg) served as the tungsten source and was placed in the central heating zone along with c‐plane sapphire substrates. The central zone was heated to 960°C and maintained for 15 min to enable film growth. Sulfur powder (S, Sigma–Aldrich, 99.99%, 3 g), pre‐solidified prior to use, was positioned upstream in a quartz boat and heated to 145°C using an external heating belt. Sulfur heating began 10 min before the center zone reached the target temperature. Prior to the growth process, the quartz tube was evacuated to base pressure and purged with a carrier gas mixture of Ar (200 sccm) and H_2_ (40 sccm) at a pressure of 20 torr. After growth, the furnace was naturally cooled to room temperature while maintaining the Ar/H_2_ flow.

Transfer of WS_2_ films was achieved through a polydimethylsiloxane (PDMS)‐assisted wet‐etching technique. A home‐prepared PDMS stamp was laminated onto the WS_2_ surface by natural adhesion. The sapphire substrate was subsequently etched away in a KOH solution, releasing the WS_2_ film onto the PDMS. The PDMS/WS_2_ assembly was then aligned and brought into contact with the freestanding metasurface, followed by vacuum treatment for 1 h to improve adhesion. Finally, gentle heating at 60°C for 10 min facilitated detachment of the PDMS, leaving the WS_2_ film successfully transferred onto the metasurface.

### 4.4 Optical Characterization

The excitation light was focused onto the sample using a 5× objective lens. Photoluminescence (PL) spectra of the freestanding metasurfaces were measured with a confocal laser microscope system (alpha300 R, WITec, Germany). A continuous‐wave (CW) laser with a wavelength of 488 nm served as the excitation source. The excitation light and the emission light were focused and collected, respectively, by objective lenses with magnifications of 5 × (NA = 0.25, corresponding to an angle within 5.7° in air). The excited emission was collected and analyzed with a spectrometer. The emission intensity distributions were obtained using a motorized *x*−*y*‐sample scanning stage for confocal emission imaging.

## Author Contributions

The manuscript was written through the contributions of all authors. All authors have given approval to the final version of the manuscript.

## Conflicts of Interest

The authors declare no conflicts of interest.

## Funding

This study was supported by JSPS KAKENHI Grant Numbers JP23K26155, JP25KF0083, and JP25H01614. Advanced Research Infrastructure for Materials and Nanotechnology in Japan (ARIM) Proposal Number JPMXP1225NM5090. Quantum Leap Flagship Program Grant Number JPMXS0118067246.

## Supporting information




**Supporting File**: smll73639‐sup‐0001‐SuppMat.pdf.

## Data Availability

The data that support the findings of this study are available in the supplementary material of this article.

## References

[1] F. Xia , H. Wang , D. Xiao , M. Dubey , and A. Ramasubramaniam , “Two‐Dimensional Material Nanophotonics,” Nature Photonics 8 (2014): 899–907, 10.1038/nphoton.2014.271.

[2] M. Turunen , M. Brotons‐Gisbert , Y. Dai , et al., “Quantum Photonics With Layered 2D Materials,” Nature Reviews Physics 4 (2022): 219–236, 10.1038/s42254-021-00408-0.

[3] T. Mueller and E. Malic , “Exciton Physics and Device Application of Two‐Dimensional Transition Metal Dichalcogenide Semiconductors,” npj 2D Materials and Applications 2 (2018): 29, 10.1038/s41699-018-0074-2.

[4] S. Wu , S. Buckley , J. R. Schaibley , et al., “Monolayer Semiconductor Nanocavity Lasers With Ultralow Thresholds,” Nature 520 (2015): 69–72, 10.1038/nature14290. 25778703

[5] D. B. Velusamy , R. H. Kim , S. Cha , et al., “Flexible Transition Metal Dichalcogenide Nanosheets for Band‐Selective Photodetection,” Nature Communications 6 (2015): 8063, 10.1038/ncomms9063. PMC4569699 26333531

[6] S. Butun , S. Tongay , and K. Aydin , “Enhanced Light Emission From Large‐Area Monolayer MoS_2_ Using Plasmonic Nanodisc Arrays,” Nano Letters 15 (2015): 2700–2704, 10.1021/acs.nanolett.5b00407. 25729895

[7] M. Koperski , K. Nogajewski , A. Arora , et al., “Single photon Emitters in Exfoliated WSe_2_ Structures,” Nature Nanotechnology 10 (2015): 503–506, 10.1038/nnano.2015.67. 25938573

[8] J. H. Chen , Y. F. Xiong , F. Xu , and Y. Q. Lu , “Silica Optical Fiber Integrated With Two‐Dimensional Materials: Towards Opto‐Electro‐Mechanical Technology,” Light: Science & Applications 10 (2021): 78, 10.1038/s41377-021-00520-x. PMC8046821 33854031

[9] T. Bucher , A. Vaskin , R. Mupparapu , et al., “Tailoring Photoluminescence from MoS_2_ Monolayers by Mie‐Resonant Metasurfaces,” ACS Photonics 6 (2019): 1002.

[10] C. W. Hsu , B. Zhen , A. D. Stone , J. D. Joannopoulos , and M. Soljačić , “Bound States in the Continuum,” Nature Reviews Materials 1 (2016): 16048, 10.1038/natrevmats.2016.48.

[11] S. I. Azzam and A. V. Kildishev , “Photonic Bound States in the Continuum: From Basics to Applications,” Advanced Optical Materials 9 (2020): 2001469, 10.1002/adom.202001469.

[12] A. Kodigala , T. Lepetit , Q. Gu , B. Bahari , Y. Fainman , and B. Kante , “Lasing action From Photonic Bound States in Continuum,” Nature 541 (2017): 196–199, 10.1038/nature20799. 28079064

[13] D. Xing , M. H. Chen , Z. Wang , et al., “Solution‐Processed Perovskite Quantum Dot Quasi‐BIC Laser From Miniaturized Low‐Lateral‐Loss Cavity,” Advanced Functional Materials 34 (2024): 2314953, 10.1002/adfm.202314953.

[14] M. Wu , L. Ding , R. P. Sabatini , et al., “Bound State in the Continuum in Nanoantenna‐Coupled Slab Waveguide Enables Low‐Threshold Quantum‐Dot Lasing,” Nano Letters 21 (2021): 9754–9760, 10.1021/acs.nanolett.1c03696. 34780696

[15] S. Romano , M. Mangini , E. Penzo , et al., “Ultrasensitive Surface Refractive Index Imaging Based on Quasi‐Bound States in the Continuum,” ACS Nano 14 (2020): 15417–15427, 10.1021/acsnano.0c06050. 33171041

[16] A. P. Anthur , H. Zhang , R. Paniagua‐Dominguez , et al., “Continuous Wave Second Harmonic Generation Enabled by Quasi‐Bound‐States in the Continuum on Gallium Phosphide Metasurfaces,” Nano Letters 20 (2020): 8745–8751, 10.1021/acs.nanolett.0c03601. 33206536

[17] T. Santiago‐Cruz , S. D. Gennaro , O. Mitrofanov , et al., “Resonant Metasurfaces for Generating Complex Quantum States,” Science 377 (2022): 991–995, 10.1126/science.abq8684. 36007052

[18] Y. L. Ho , C. F. Fong , Y. J. Wu , et al., “Finite‐Area Membrane Metasurfaces for Enhancing Light‐Matter Coupling in Monolayer Transition Metal Dichalcogenides,” ACS Nano 18 (2024): 24173–24181, 10.1021/acsnano.4c05560. 39167162

[19] F. L. Hsieh , C. Z. Deng , S. K. Huang , et al., “Large‐Area Photonic Membranes Achieving Uniform and Strong Enhancement of Photoluminescence and Second‐Harmonic Generation in Monolayer WSe_2_ ,” Small Methods 10 (2026): 01693, 10.1002/smtd.202501693. 41630151

[20] C. Z. Deng , C. H. Chiang , S. Shi , et al., “Atomic‐Scale Light Coupling Control in Ultrathin Photonic Membranes,” Advanced Functional Materials (2026): 24286, 10.1002/adfm.202524286.

[21] N. Bernhardt , K. Koshelev , S. J. U. White , et al., “Quasi‐BIC Resonant Enhancement of Second‐Harmonic Generation in WS_2_ Monolayers,” Nano Letters 20 (2020): 5309–5314, 10.1021/acs.nanolett.0c01603. 32530635

[22] J. Lee , M. Jeong , J. Jang , et al., “Bound‐States‐in‐the‐Continuum‐Induced Directional Photoluminescence With Polarization Singularity in WS_2_ Monolayers,” Nano Letters 25 (2025): 861–867, 10.1021/acs.nanolett.4c05544. 39760918

[23] I. Barth , M. Deckart , D. Conteduca , et al., “Lasing From a Large‐Area 2D Material Enabled by a Dual‐Resonance Metasurface,” ACS Nano 18 (2024): 12897–12904, 10.1021/acsnano.4c00547. 38710615 PMC11112975

[24] F. J. F. Löchner , A. George , K. Koshelev , et al., “Hybrid Dielectric Metasurfaces for Enhancing Second‐Harmonic Generation in Chemical Vapor Deposition Grown MoS_2_ monolayers,” ACS Photonics 8 (2020): 218.

[25] E. Maggiolini , L. Polimeno , F. Todisco , et al., “Strongly Enhanced Light–Matter Coupling of Monolayer WS_2_ From a Bound State in the Continuum,” Nature Materials 22 (2023): 964–969, 10.1038/s41563-023-01562-9. 37217703

[26] V. Kravtsov , E. Khestanova , F. A. Benimetskiy , et al., “Nonlinear Polaritons in a Monolayer Semiconductor Coupled to Optical Bound States in the Continuum,” Light: Science & Applications 9 (2020): 56. 10.1038/s41377-020-0286-z PMC7145813 32284858

[27] T. Weber , L. Kuhner , L. Sortino , et al., “Intrinsic Strong Light‐Matter Coupling With Self‐Hybridized Bound States in the Continuum in Van Der Waals Metasurfaces,” Nature Materials 22 (2023): 970–976, 10.1038/s41563-023-01580-7. 37349392 PMC10390334

[28] L. Xu , K. Zangeneh Kamali , L. Huang , et al., “Dynamic Nonlinear Image Tuning Through Magnetic Dipole Quasi‐BIC Ultrathin Resonators,” Advanced Science 6 (2019): 1802119, 10.1002/advs.201802119. 31406659 PMC6685498

[29] Z. F. Sadrieva , I. S. Sinev , K. L. Koshelev , et al., “Transition From Optical Bound States in the Continuum to Leaky Resonances: Role of Substrate and Roughness,” ACS Photonics 4 (2017): 723–727, 10.1021/acsphotonics.6b00860.

[30] A. Leitis , M. L. Tseng , A. John‐Herpin , Y. S. Kivshar , and H. Altug , “Wafer‐Scale Functional Metasurfaces for Mid‐Infrared Photonics and Biosensing,” Advanced Materials 33 (2021): 2102232, 10.1002/adma.202102232. 34494318 PMC11468586

[31] M. Hirler , A. A. Antonov , E. Bau , et al., “Accessible, All‐Polymer Metasurfaces: Low Effort, High Quality Factor,” ACS Nano 20 (2026): 6722–6731, 10.1021/acsnano.5c15415. 41708058 PMC12961952

[32] J. Wang , J. Kühne , T. Karamanos , C. Rockstuhl , S. A. Maier , and A. Tittl , “All‐Dielectric Crescent Metasurface Sensor Driven by Bound States in the Continuum,” Advanced Functional Materials 31 (2021): 2104652, 10.1002/adfm.202104652.

[33] W. Adi , S. Rosas , A. Beisenova , et al., “Trapping Light in Air With Membrane Metasurfaces for Vibrational Strong Coupling,” Nature Communications 15 (2024): 10049, 10.1038/s41467-024-54284-0. PMC11579285 39567485

[34] D. R. Southworth , R. A. Barton , S. S. Verbridge , et al., “Stress and Silicon Nitride: A Crack in the Universal Dissipation of Glasses,” Physical Review Letters 102 (2009): 225503, 10.1103/PhysRevLett.102.225503. 19658878

[35] S. Schmid , K. D. Jensen , K. H. Nielsen , and A. Boisen , “Damping Mechanisms in High‐ Q Micro and Nanomechanical String Resonators,” Physical Review B 84 (2011): 165307, 10.1103/PhysRevB.84.165307.

[36] C. Acikgoz , M. A. Hempenius , J. Huskens , and G. J. Vancso , “Polymers in Conventional and Alternative Lithography for the Fabrication of Nanostructures,” European Polymer Journal 47 (2011): 2033–2052, 10.1016/j.eurpolymj.2011.07.025.

[37] V. C. Su , C. H. Chu , G. Sun , and D. P. Tsai , “Advances in Optical Metasurfaces: Fabrication and Applications [Invited],” Optics Express 26 (2018): 13148, 10.1364/OE.26.013148. 29801344

[38] Y. Chen , “Nanofabrication by Electron Beam Lithography and Its Applications: A Review,” Microelectronic Engineering 135 (2015): 57–72, 10.1016/j.mee.2015.02.042.

[39] M. Iwanaga , X. Yang , V. Karanikolas , T. Kuroda , and Y. Sakuma , “Prominently Enhanced Luminescence From a Continuous Monolayer of Transition Metal Dichalcogenide on All‐Dielectric Metasurfaces,” Nanophotonics 13 (2024): 95–105, 10.1515/nanoph-2023-0672. 39633992 PMC11501220

[40] W. He , S. Wan , Y. Zuo , et al., “Loss‐Enabled Chirality Inversion in Terahertz Metasurfaces,” Physical Review Letters 134 (2025): 106901, 10.1103/PhysRevLett.134.106901. 40153642

[41] Z. Yu , W. He , S. Hu , et al., “Creating Anti‐Chiral Exceptional Points in Non‐Hermitian Metasurfaces for Efficient Terahertz Switching,” Advanced Science 11 (2024): 2402615, 10.1002/advs.202402615. 38757557 PMC11267315

