# Supplementary material for: Freestanding Polymer Metasurface Supporting Higher‐Order Optical Resonances for Strong Field Enhancement in TMD Monolayers
Source: Small. 2026 May 17;22(35):e13320. doi: 10.1002/smll.202513320 (PMC13288818; doi:10.1002/smll.202513320)
Supplement: Supplementary file 1 — Supporting File: smll73639‐sup‐0001‐SuppMat.pdf. [file SMLL-22-e13320-s001.pdf]

Supporting Information

# Freestanding Polymer Metasurface Supporting Higher-Order Optical Resonances for Strong Field Enhancement in TMD Monolayers

*Chih-Zong Deng*[1], *Sunhao Shi*[2], *Chun-Hao Chiang*[1], *Mu-Hsin Chen*[1], *Shuaicheng Liu*[3], *Haruyuki Sakurai*[4], *Jui-Han Fu*[2], *Kuniaki Konishi*[4], *Masanobu Iwanaga*[1], *Vincent Tung*[2], *and Ya-Lun Ho**[1]

[1]Research Center for Electronic and Optical Materials, National Institute for Materials Science (NIMS), 1-1 Namiki, Tsukuba, Ibaraki 305-0044, Japan

[2]Department of Chemical System Engineering, Graduate School of Engineering, The University of Tokyo, 7-3-1 Hongo, Bunkyo, Tokyo 113-8656, Japan

[3]Department of Physics, Graduate School of Science, The University of Tokyo, 7-3-1 Hongo, Bunkyo, Tokyo 113-0033, Japan

[4]Institute for Photon Science and Technology, Graduate School of Science, The University of Tokyo, 7-3-1 Hongo, Bunkyo, Tokyo 113-0033, Japan

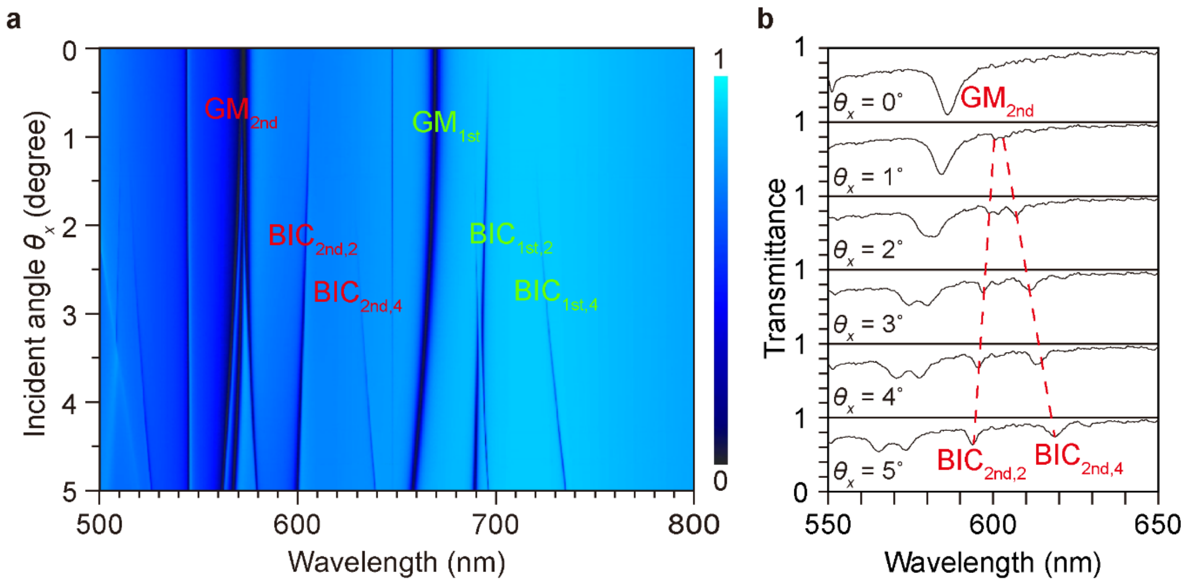


**Figure S1.** a) Simulated and b) experimental angle-resolved transmittance spectra under *x*-polarized illumination along the *x*-direction, showing GMs and quasi-BICs.

Figure S1 presents the a) simulated and b) experimental transmittance spectra of the freestanding metasurface with lattice period $P$ = 560 nm, hole diameter $D$ = 280 nm, and thickness $T$ = 400 nm on a 50 nm-thick SiN membrane as a function of incident angle $\theta_x$. This narrow angular range is chosen to isolate symmetry-protected bound states in the continuum (BICs), which are expected to occur at the Γ point. At normal incidence ($\theta$ = 0°), the spectrum exhibits optical modes with theoretically infinite Q-factors—a defining characteristic of ideal BICs—enabling clear differentiation from guided modes. The spectra show that the $BIC_{1st,2}$ and $BIC_{2nd,2}$ are accessible under *x*-tilted illumination ($\theta_x$).

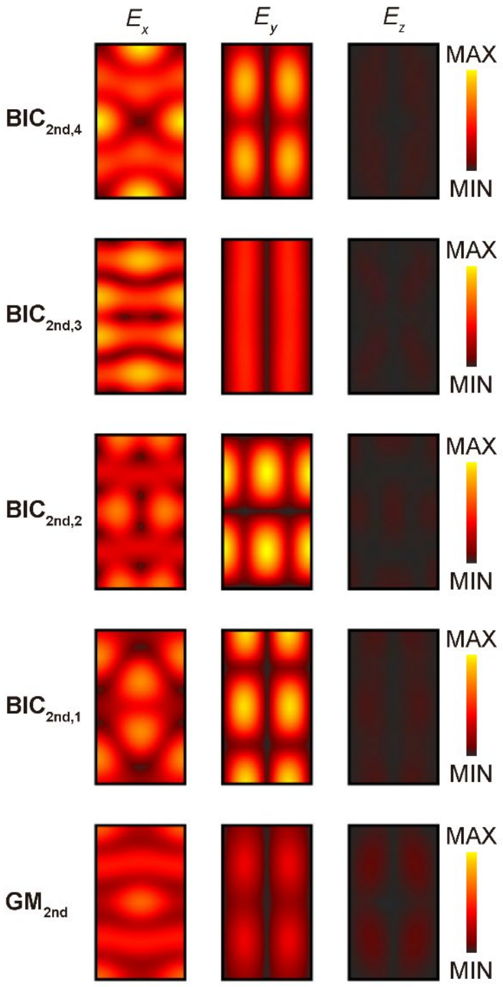


**Figure S2.** Simulated distributions of the *x*-, *y*- and *z*-components of the electric field of the first-order quasi-BICs and GM for a freestanding metasurface with a lattice period $P$ = 560 nm. The three components are noted as $E_x$, $E_y$, and $E_z$, respectively.

Figure S2 presents the electric field distributions of the first-order quasi-BICs and GM. The resonances are identified as TE-modes as the electric fields are dominated by the in-plane components $E_x$ and $E_y$.

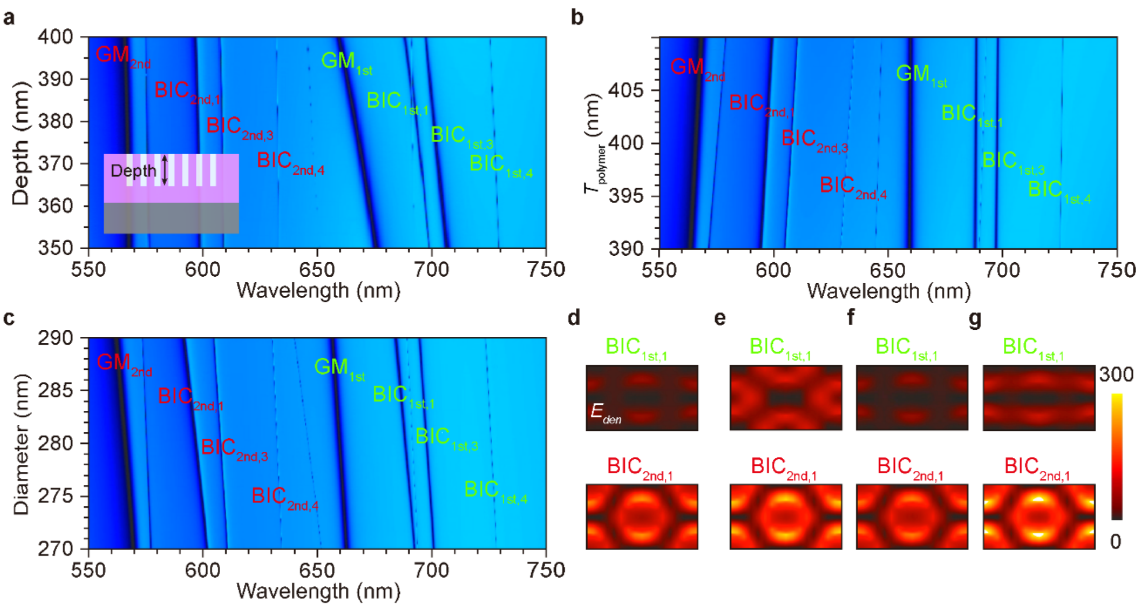


**Figure S3** Influence of fabrication imperfections on the freestanding metasurface. a–c) Sensitivity analysis of a) air-hole depth, b) polymer thickness ($T_{polymer}$), and (c) hole diameter ($D$). The electric energy density distributions in the *xy*-plane at the top surface are shown for the $BIC_{1st,1}$ and $BIC_{2nd,1}$. d) Ideal structure with air-hole depth = 400 nm, $T_{polymer}$ = 400 nm, and $D$ = 280 nm. e) Reduced air-hole depth of 350 nm. f) Reduced polymer thickness $T_{polymer}$ = 390 nm. g) Reduced hole diameter $D$ = 270 nm. The incident light is *x*-polarized and impinges at an angle of 2.5° along the *y*-direction.

Figure S3 illustrates the influence of fabrication imperfections, including air-hole depth, polymer thickness, and hole diameter. A comparison of these parameters highlights the distinct sensitivities of first-order and second-order modes. The second-order modes utilized in this study offer higher structural robustness compared to their first-order counterparts, particularly regarding variations in air-hole depth. Specifically, a reduction in air-hole depth from the designed 400 nm to 375 nm results in a resonance shift of 28.79 nm for the first-order GM, whereas the second-order GM shifts by only 1.37 nm. A similar trend is observed for the BICs, where the first-order BIC shifts by 6.18 nm compared to a mere 1.47 nm for the second-order BIC. This disparity arises because the electric field of first-order modes is primarily confined within the SiN layer, making them highly sensitive to vertical geometry. In contrast, the Q-factors for both mode orders remain relatively stable, within a 10% variation range.

Regarding the polymer thickness, both first- and second-order modes exhibit low sensitivity to thickness fluctuations. A deviation from the designed 400 nm to 390 nm results in resonance shifts of less than 2 nm and Q-factor variations within 10% for both BICs and GMs. Given that the polymer thickness is large relative to the operating wavelength and that the spin-coating process maintains stability within a typical +/- 10 nm range, these variations do not significantly disturb the optical modes.

Finally, the analysis of hole diameter reveals that while resonance wavelength shifts remain modest (less than 5 nm when comparing the 280 nm design to a 270 nm result), the Q-factor is highly sensitive due to the symmetry-protected nature of the BICs. Reducing the hole diameter increases the Q-factors of the first-order and second-order BICs by 52% and 35%, respectively, while the GM shows a negligible change of only 3%. This increase in Q-factor directly correlates to stronger field enhancement at the top surface, thereby serving as a primary driver for the enhanced PL factors observed experimentally.

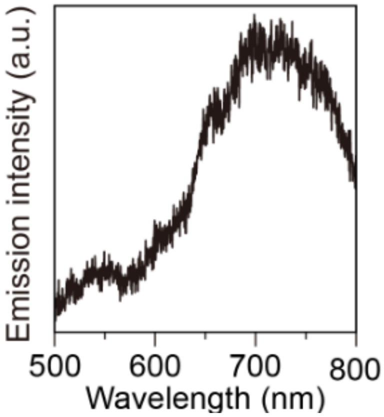


**Figure S4** PL spectrum of the 50-nm-thick unstructured SiN membrane. The unstructured SiN membrane was illuminated with a 488 nm continuous-wave laser, generating a broadband PL emission spanning approximately 500–800 nm, attributed to defect-related states in SiN.

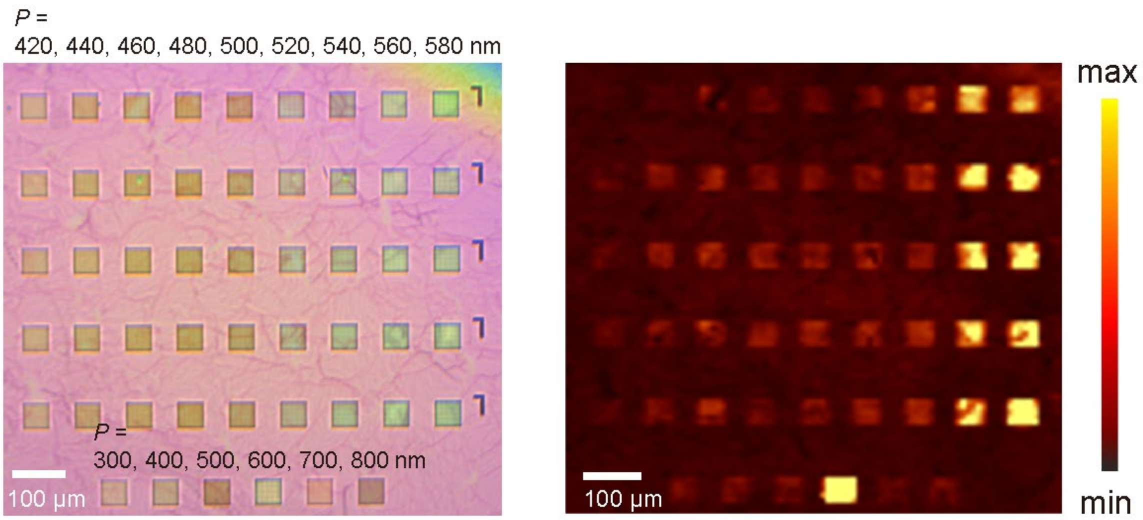


**Figure S5** OM images of the freestanding metasurfaces and large-area PL mapping of the membrane including hole-array pattern and unstructured regions for the wavelength = 620 nm ($WS_2$ exciton emission peak).

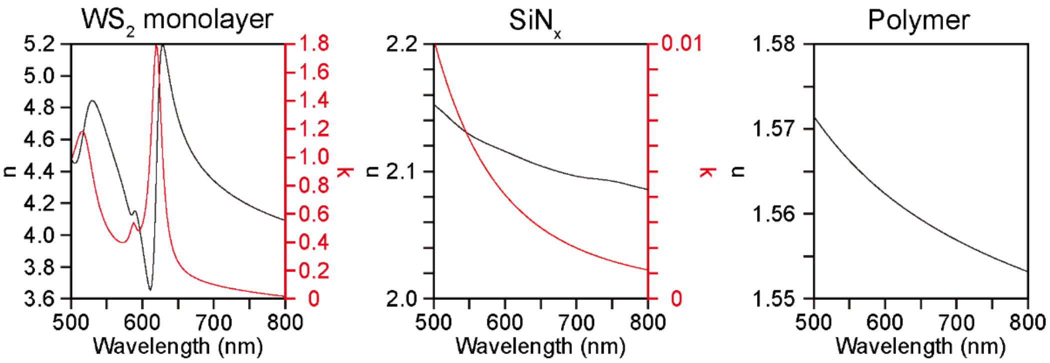


**Figure S6** Refractive index of $WS_2$ monolayer, SiN, and polymer (CSAR 62). The complex refractive indices (n, k) for the $WS_2$ monolayer, SiN membrane, and polymer resist were determined using a spectroscopic ellipsometer (M-2000 Ellipsometer, J.A. Woollam, USA). Measurements were performed at an angle of incidence of 65˚ with 40 accumulations per data point to ensure a high signal-to-noise ratio. The experimental data were analyzed using a General Oscillator (Gen-Osc) model within the CompleteEASE software environment.

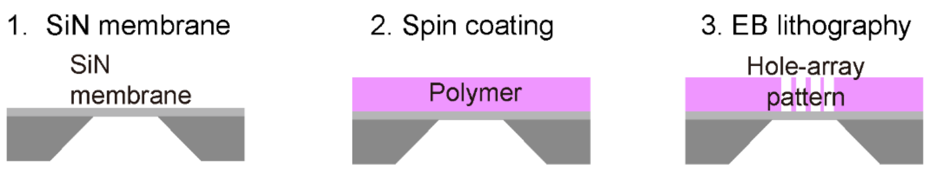


**Figure S7** Fabrication process of freestanding metasurface.

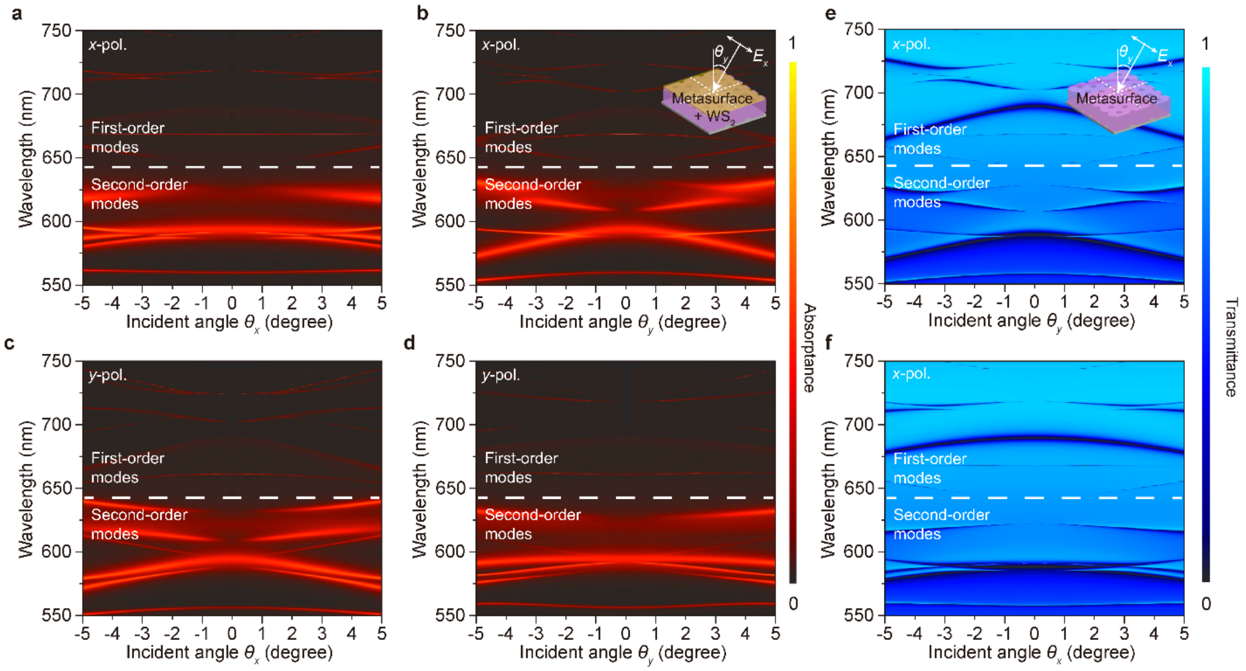


**Figure S8** a-d) Simulated absorptance of the $WS_2$-transferred membrane metasurface under different polarizations and incident angles. e, f) Simulated transmittance of the bare membrane metasurface for *x*-polarized light with incidence along the *x*- and *y*-directions.
